# Epigenetics, epistasis and epidemics

**DOI:** 10.1093/emph/eot009

**Published:** 2013-04-22

**Authors:** Fernando Baquero

**Affiliations:** Department of Microbiology, Ramón y Cajal University Hospital, Ramón y Cajal Institute for Health Research (IRYCIS), Madrid, Spain

**Keywords:** epigenetics, epistasis, epidemiology, antibiotic resistance



We are already installed in the post-genetic or meta-genetic search for biological causalities. Until recently causation in biology was almost universally attributed to the main genetic factor, the gene, with a close correspondence with the encoded character, the phenotype. The term ‘epigenetics’ refers to studies ‘above the gene’ and refers to heritable (reproducible) changes in gene function that cannot be explained by mutations in DNA sequence. The term ‘epistasis’ etymologically means the ‘act of stopping’ (any ‘on-off’ action) and refers to the phenomenon in which one or more genes influences the function of others. The term ‘epidemics’ (in our case, bacterial epidemics) means ‘what is upon the people’ and refers to the consequences of the action of bacterial organisms beyond the individual, that is, on the community. The recent publication of Borrell *et al.* [1] in *Evolution, Medicine and Public Health* helps us to consider how a public health problem (the spread of extensively drug-resistant tuberculosis) might depend not only on the emergence of mutational adaptive traits but also require ‘over the gene’ interactions, concerted actions of various mutated non-allelic genes. Over-the-gene adaptive advantages of bacterial pathogens occur by epigenetic and epistatic interactions influencing many susceptible individuals (epidemics). In this ‘epi-’ perspective, not a single type of element, not a single cell, not a single gene, not a single individual creates the public health problem. As these multiple interactions are to a certain extent of stochastic nature, the complexity of the causal analysis increases significantly, leading to what might be qualified as ‘causal relativity’ or, in general ‘biological relativity’ [2].

Epigenetics might influence the evolution of antibiotic resistance. Stochastic variation in the expression of sets of genes is expected to occur even in isogenic populations, due to factors that include DNA methylation, covalent modification of DNA-binding proteins, non-coding DNA or RNA splicing factors. The hypothesis is that these factors, by influencing stochastic fluctuations in cellular components, produce epigenetic variation, and consequently phenotypic diversity for natural selection to act upon. Antibiotic exposure will select cells with gene expression patterns providing a resistance phenotype. Under these circumstances, the number of cells able to survive in the presence of a drug might be sufficient to evolve more effective mutational mechanisms of resistance [3]. However, it is not always easy to differentiate canonical factors involved in epigenetics from other ‘random-and-reversible’ adaptive mechanisms such as transient gene amplification, a very frequent ‘genetic’ mechanism for increased antibiotic resistance not implying stable changes in the gene sequence [4]. In a wider and more eclectic view, epigenetics can be defined as the study of cell lineage formation by non-mutational mechanisms.

Epistasis (*ε*) is defined as ε*_ij_* = *W_ij_ − W_i_ · W_j_*, where *W*_0_ (implicit) is wild-type fitness, *W_ij_* fitness when the *i* and *j* traits are combined, and *W_i_ · W_j_* corresponds to the null expectation (as if *i* and *j* were independent components of the total fitness). Considering *W*_0_ = 1, a positive sign result indicates beneficial epistasis (*W_ij_* > *W*_0_), or sign epistasis [5]. The work of Borrell *et al.* [1] shows a clear example of sign epistasis acting on the evolution of antibiotic resistance. They document that *Mycobacterium* isolates carrying particular mutations conferring resistance to two different antibiotics (rifampicin and ofloxacin) have higher fitness than corresponding strains carrying only one of these mutations, and in some cases also higher than the original wild strain. This occurs even if strains with single mutations exhibit reduced fitness in comparison with the wild-type ancestor (but they should in any case have a tolerable fitness, above a ‘minimal fitness for epistasis’). In short, the genetic background (harboring one or the other mutation) determines the adaptive weight or the other one. These epistatic interactions have probably greatly influenced the molecular evolution of both genomes and proteins [6].

Borrell *et al.* [1] detect sign epistasis by using competitive fitness experiments in an appropriate culture media. Fitness refers to ability of organisms (genotypes) to survive and reproduce in an appropriate culture medium where they are studied (*in vitro* fitness) or find themselves in nature (*in natura* fitness). The consequence of this survival and reproduction is that organisms contribute genes to the next generation. Most frequently, fitness is expressed as ‘relative fitness’, comparing fitness of a variant genotype with that of the wild genotype. There has been a long-lasting discussion about the relevance of *in vitro* systems (relative growth rates, competition experiments in co-cultures) to predict *in natura* behavior of microorganisms. Of course the environmental conditions change dramatically from a culture tube, where we are measuring ‘fitness-under-optimal growth conditions’ (*W*_OG_) to an infective site. However, we can consider that under *in vitro* optimal conditions for growth, the microbial biosynthetic machinery is acting at full speed, and that should maximize the possibilities of detecting failures, like an aircraft forcing the engines before taking off. The assumption is that these failures might also impose a biological cost in any other conditions, but obviously the possibility of ‘adaptive failures’ that might be only detectable under precise circumstances cannot be excluded. The results from Borrell *et al.* [1] indicate that in the case of multi-resistant *Mycobacterium*, the *in vitro* predicted fitness of the combinations of particular allelic forms of antibiotic-resistance mutations correlates with the fitness observed *in natura* (high frequency of these combination of mutations in widespread *Mycobacterium tuberculosis* isolates in South Africa).

Interestingly, the results of Borrell *et al.* [1] illustrate that, above a threshold of minimal fitness, epistatic effects between allelic forms of *gyrA* (ofloxacin-resistance) and *rpoB* (rifampicin-resistance) will be stronger when the individual mutations are associated with large fitness defects. That is, unexpected fitness gains arise from the interaction of mutations that independently reduce fitness. In other words, neutral or deleterious (above a threshold) genetic changes might influence the topology of the interactive space of genotypes, and ultimately be critical in reaching an optimal phenotype. In fact this observation highlights the potency of ‘evolution behind the curtain’. Of course the successful combination of mutations occurs stochastically, and a typical feature of epistasis is illustrated by this non-linear, unexpected system-level behavior arising from combinations of components working together [5]. In a sense, thanks to a stressful exposure (antimicrobial drugs), there is a possibility that the bacterial population improves its fitness, even in the absence of antibiotics [7]. That is an excellent example of the evolutionary acceleration that rugged adaptive landscapes might impose on organisms. In fact, antibiotic exposure and antibiotic resistance shape the evolutionary rate and, in general, influence the biology of bacteria at multiple levels, from genes right up to the population of organisms [8].

Even though the Borrell et al. publication [1] is focused on the epistatic effects influencing antibiotic resistance and overall *Mycobacterium* fitness in extensively drug-resistant tuberculosis, their observations are of general significance in the understanding of the emergence of microbial-related public health problems. We are obliged to consider that the genetic and phenotypic variability of bacterial organisms is extremely complex not only in bacterial species but also in bacterial clones and strains. Local selective events tend to purge such high diversity, but variability is always efficiently recovered via *ex unibus plurum* dynamics guided by diversifying attractors, conditions favoring diversification as an adaptive evolutionary configuration [9]. Such heterogeneity among individuals serves as a bet-hedging (risk-spreading) mechanism for the population. Bet-hedging populations maximize long-term survival in changing environments as they contain lineages expressing non-optimal phenotypes today that might be optimal tomorrow [10]. For instance, persistence, a high-risk investment for a fraction of the bacterial population (slow or non-growth), assures the maintenance of the organism under antibiotic exposure.

Our view is that epigenetics and epistasis are to a certain extent equivalent mechanisms, with a lower degree of heritability than mutations in DNA sequences, but shaping the population biology of bacterial organisms in an efficient way. Up to a point, epigenetics, epistasis, mutational events and introgressive horizontal gene transfer interact synergistically to ensure inclusive fitness for the bacterial populations. We could imagine a multi-dimensional interactive network-space combining epigenetic, epistatic, mutational and introgressive effects, where evolving bacteria continuously scan variable adaptive landscapes. From the point of view of Public Health, human or animal populations constitute the target landscape for bacterial exploitation; at their turn, these populations are composed of a multiplicity of variant individuals with different susceptibilities and variable chances to be affected by the bacterial infective processes. Epidemiology must deal with the study of such ‘target’ complexity. The coincidence of an appropriate adaptive combination in the microorganism with an appropriate host landscape will result in the emergence of epidemic bacterial spread. It is our conviction that future Public Health Microbiology [11] should in the near future provide a very broad, integrative platform of basic and theoretical research that integrates the findings in epigenetics, epistasis, mutation, horizontal gene transfer, epidemiology and evolution to understand the complex causality and to control the complex interactions of humans, animals and the Microbiosphere.

## Funding

The author’s work has been sponsored by grants from the European Union (PAR-241476 and EvoTAR-282004) and the Instituto de Salud Carlos III of Spain (FIS-PI10-02588).

**Conflict of interest**: none declared.
